# Synthesis, spectral characterization, and theoretical investigation of the photovoltaic properties of (*E*)-6-(4-(dimethylamino)phenyl)diazenyl)-2-octyl-benzoisoquinoline-1, 3-dione

**DOI:** 10.1186/s13065-022-00896-w

**Published:** 2022-12-03

**Authors:** Mbang I. Ofem, Hitler Louis, John A. Agwupuye, Umar S. Ameuru, Gloria C. Apebende, Terkumbur E. Gber, Joseph O. Odey, Neksumi Musa, Ayi A. Ayi

**Affiliations:** 1 Department of Chemistry, Faculty of Physical Sciences, Cross River University of Technology, Calabar, Nigeria; 2 Computational and Bio-Simulation Research Group, University of Calabar, Calabar, Nigeria; 3 Department of Pure and Applied Chemistry, Faculty of Physical Sciences, University of Calabar, Calabar, Nigeria; 4 Department of Polymer and Textile Engineering, Ahmadu Bello University, Zaria, Nigeria; 5 Department of Environmnetal Sciences, Sharda University, Greater Noida, India; 6 Inorganic Materials Research Laboratory, Department of Pure and Applied Chemistry, Faculty of Physical Sciences, University of Calabar, Calabar, Nigeria

**Keywords:** Benzeneisoquinolinedione, synthesis, Characterization, Photovoltaic, DFT, TD-DFT

## Abstract

This research work focuses on the synthesis, characterization through spectra (FT-IR, UV–vis, and ^1^H-NMR) investigations, and the use of density functional theory (DFT) along with time-dependent density functional theory (TD-DFT) to investigate the electronic, structural, reactivity, photophysical properties, and the photovoltaic properties of a novel (*E*)-6-(4-(dimethylamino)phenyl)diazenyl)-2-octyl-benzoisoquinoline-1,3-dione. The structure of the synthesized compound was modeled using the Gaussian09W and GaussView6.0.16 softwares employing B3LYP and 6–31 + G(d) basis set. The DFT studies was performed in order to investigate the Frontier Molecular Orbital (FMO), Natural Bond Orbital (NBO), charge distribution, Nonlinear Optics (NLO), and stability of the titled molecule. The HOMO–LUMO energy gap which corresponds to the difference between HOMO and LUMO energies of the studied compound was found to be 2.806 eV indicating stiff and smooth nature of the titled molecule. This accounts for the less stability and high chemical reactivity of the compound. The photovoltaic properties were conducted to evaluate the light harvesting efficiency (LHE), short circuit current density (J_SC_), Gibbs free energy of injection ($\Delta {G}_{inj}$), open cycled voltage (V_OC_) and Gibbs free energy regeneration ($\Delta {G}_{reg}$) and solar cell conversion efficiency. Interestingly, the results obtained were found to be in good agreement with other experimental and computational findings.

**Supplementary Information:**

The online version contains supplementary material available at 10.1186/s13065-022-00896-w.

## Introduction

The relationship between the azo compounds and their electronic, structural, reactivity, photophysical and photovoltaic properties, which are generally synthesized by diazotization reaction using a primary aromatic amine containing one or more nucleophiles that are very important because of their delocalized electrons are widely employed for applications in drugs manufacturing, cosmetics and textile industries and in material science [1, 2]. These azo compounds are the largest and most versatile class of dyes; they possess intensive bright colours ranging from orange to yellow, crimson red, blue and even green depending on the structure of the molecule. These molecules undergo a fast intramolecular charge transfer (ICT) upon light excitation. Aside their characteristics colouring functions, azo compounds are popular for their therapeutic uses such as antineoplastic, antioxidant, analgesic, anti-inflammatory, antiviral, and antitumor activities [3–5]. The azo compound under excited state are stable to light absorption and electronic excitation are characteristics features of the azo core as many azo polymers are highly photoresponsive and photoinduced along with applications in dye sensitizing solar cells, electro-optical activities and charged separation represents one of the main properties of these molecules making them suitable for the wide range of applications [6, 7].

Azo compounds are widely used in the field of material science and pharmaceuticals, this is because it contains one or more azo bonds (− N = N−) as a chromophore group in connection with aromatic structures containing functional groups such as halogens, hydroxyl group and sulphate group. Studies have shown that many devices have been successfully created using these organic molecules as building blocks. Light-Emitting Azo-dyes (LEAs), Organic Photovoltaic Cells (OPVCs), Azo-dye Sensitizing Solar Cells (ASSCs) [8, 9] and some luminescent solar concentrators (LSCs) are some of the potential applications.

Many researchers have used Density Functional Theory (DFT) in recent years to examine photovoltaic and photophysical properties. Babu, N et al. [8] conducted theoretical studies of the optoelectronic and photovoltaic properties of D-A polymer monomers using Density Functional Theory (DFT) and reported that the calculated band gap Eg of the monomers considered increases 3,6-MMCB-OCP ≈ 3,6-MMCB-BCO < 3,6-MMCB-SDP < 3,6-MMCB-SCP < 3,6-MMCB-TCP < 3,6-MMCB-TDP < 3,6-MMCB-BCS < 3,6-MMCB-BCT in both in the gas and solvent phases. Yeşilda, A et al. [9] investigated the synthesis of benzidine-based conjugated organic materials bearing donor–acceptor groups: DFT studies and photovoltaic applications, and their results show that the power conversion efficiency (PCE) values for compounds 2, 4a-b were calculated as 2.25, 2.70, and 2.80%, respectively. In comparison to compounds 2 and 4a, dipyrenyl-bearing benzidine derivative 4b demonstrated a higher redshift in the absorption wavelength with a noteworthy high PCE value and a low Eg value, which can be attributed to 4b's increased stability due to the longer pi conjugation. In the same vein, Vuai, S. A [10] investigated DFT and TD-DFT studies for optoelectronic properties of coumarin-based donor pi acceptor dyes: applications in dye-sensitized solar, and their findings revealed that the LUMO energies of D1-CM-A1, D2-CM-A2, D3-CM-A3 and D4-CM-A4 were higher than the conduction band edge of TiO_2._ Güngördü et al. [11] investigated the photovoltaic performance attributes, DFT analyses, and synthesis of (E)-3-(diphenxy) acrylic acid substituted phthalocyanine complexes and discovered that the complexes’ predicted power conversion efficiencies (%) were at an appreciable level.

A comprehensive photophysical and photovoltaic study of the azo compound (E)-6-(4-(dimethylamino)phenyl)diazinyl)-2-octyl-benoisoquinoline-1,3-dione is given in this paper. This molecule is unique in that it is a conjugated system with a donor–acceptor-donor (D-A-D) framework in which an electron-poor benzoisoquinoline-1, 3-dione core is linked to two electron-rich diazenyl rings. The initial stage in creating a series of derivatives with improved electro-optical features is accurate photophysical and photovoltaic characterization. The most often used parameter to compare the performance of one sensitizing solar cell to another is efficiency [9]. The inclusion of functional groups around the azo-dye core will be critical to improving photophysical and photovoltaic characteristics (molar absorption coefficient, quantum yield, and lifetime) and tuning the maximum emission wavelength as a function of application. In various organic solvents, particularly DMSO, the dye has a specified high molar extinction coefficient, a high photoresponsive quantum yield (varying from 0.69 to 0.9), and a very large stokes shift (greater than 4600 cm-1). The molecule under investigation was completely synthesized experimentally and theoretically validated by FT-IR, NMR, and UV–Vis spectroscopic methods. The experimental results were compared to theoretical data derived from quantum chemical computations. Density Functional Theory (DFT) at the Becke's 3-parameters combined with Lee–Yang–Parr correlation functional (B3LYP) with a 6-31G(d)basis set to do the computational quantum calculations.

The DFT calculations were effective in the investigation of similarities between the geometric and electronic properties of the compound and the study data obtained comparatively agreed with the experimental values. The Frontier molecular orbitals energies between the highest occupied molecular orbitals (HOMO) and lowest unoccupied molecular orbitals (LUMO) were analyzed to determine the reactivity and stability of the compound. Nonlinear optics (NLO) was carried out to ascertain the optical activity of the compound. Azo-dye compounds and some organic molecules possess (NLO) properties in the presence of π-conjugated molecules characterized by a donor–acceptor-donor (D-A-D) framework. The Natural Bond Orbital (NBO) analysis was also carried out to interpret the hybridization, resonance, donor–acceptor interactions and stability of the compound. The electrostatic potential (ESP) was also plotted to provide graphical representation of the chemical active sites and the reactivity.

## Experimental method

### Synthesis of 4-nitro-1, 8-naphthalic anhydride

Nitroacenaphthene (23.87 g, 0.25 mol) was dissolved in hot acetic acid (238.75 cm^3^), sodium dichromate (148.5 g) was added for 2 h at 63–70 °C. The solution was warmed gradually to 96–100 °C for 36 min and further refluxed for 4 h. The contents were washed out with hot water (500 cm^3^) cooled, filtered and the solid was washed with dilute HCl. The solid was boiled with 250 cm^3^ of 5% Na_2_CO_3_ solution for 30 min and filtered. The filtrate was acidified and the separated crystals was dried at 120 °C for 3 h to obtain 4-nitro-1,8-naphthalic anhydride, which was recrystallized from HNO_3_ (d.1.38 g/ml) to afford colourless needles [10].

### Synthesis of 4-nitrodecyl-1, 8-naphthalimid

A suspension of 4-nitro-1, 8-naphthalic anhydride (0.02 mol, 4.84 g) and decylamine (0.03 mol, 4.69 g) was stirred under reflux for 6 h in ethanol (102 cm^3^). The cooled suspension was filtered and recrystallized with ethanol.

### Synthesis of 4-Amino-n-decyl-1, 8-naphthalimide

A mixture of 4-nitro-n-decyl-1,8-naphthalimide (16 mmol, 6.78 g) and stannous chloride (90 mmol, 20.24 g) in ethanol (81 cm^3^) was refluxed for 1 h with dilute hydrochloric acid till the reaction was complete. The mixture was transferred into 100 cm.^3^ of water and the observed precipitate was filtered. The crude product was purified by recrystallization from ethanol as orange crystals [11]

### General procedure for diazotization

Dry sodium nitrite (1.4 mmol, 0.104 g) was slowly added with constant stirring to conc. H_2_SO_4_ (98%, 1.1 cm^3^) at below 10 °C. The temperature of the reaction mixture was increased to 64 °C using water bath until all the sodium nitrite was dissolved. The solution was then cooled to 0–5 °C and a mixture of 10 cm^3^ propionic acid and acetic acid (1.5:8.5 v/v) was added drop-wise with constant stirring and the temperature was raised to 10–15 °C. The finely ground powder of 4-amino-n-substituted-1,8-naphthalimide (1.5 mmol) was added portion-wise and stirring was continued for 3 h, the clear diazonium salt solution obtained was used immediately in coupling reaction [11].

### Coupling reactions

**Fig. 1:**
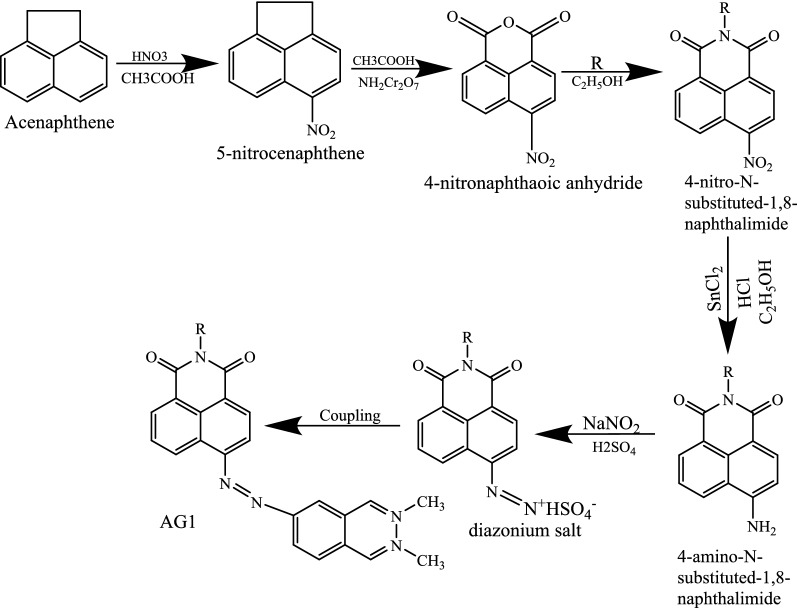
Synthesis of (E)-6-(-4(dimethylamino)phenyl)diazenyl)-2-Octyl-benzoisoquinoline-1,3-dione (AGI)

The coupling component, 2, 3-dihydroxynaphthalene was dissolved in ethanol with few drops of acetic acid added at 0–5 °C. The previously prepared diazonium solution was added drop wise over 30–40 min period with vigorous stirring. The mixture was stirred further for 2 h under 0–5 °C and pH of the solution was adjusted to 4–5 using 10% sodium acetate and then stirred further for 1 h. The resulting product was then collected by filtration, washed with warm water and then cold water and dried. The crude product was purified by recrystallizing from dimethyl sulfoxide (DMSO) several times. The reaction scheme, which is in agreement with literature report [10] is shown in Fig. 1.

### Spectral characterization of the synthesized dye

The absorption spectrum of benzoisoquinolinedione was measured in various solvents. The wavelengths of maximum absorption (λ_max_) and molar extinction coefficient ranged from 515–535 nm and 1.59 × 10^4^–3.00 × 10^4^ Lmol^−1^ cm for the synthesized dye in DMF. The λ_max_ shifts in different solvents are due to solvatochromic effects which is due to changes in the dielectric constant of the solvent. The Infrared spectrum was recorded on a Perkin-Elmer spectrum RX1 FT-IR spectrometer. ^1^HNMR spectrum was obtained on a 300 MHz Bruker instrument using deuterated chloroform (CDCl_3_) and dimethylsulphoxide (DMSO-d_6_) as solvent. Chemical shifts are reported in parts per million (ppm) downfield from the internal tetramethylsilane (TMS) [11].

### Computational details

Geometry optimization of (E)-6-(-4(dimethylamino)phenyl)diazenyl)-2-octyl-benzoisoquinoline-1,3-dione has been achieved through Density Functional Theory (DFT) method at B3LYP/6-31G(d) basis set level of approximation using Gaussian 09 W and GaussView 6.0.16 software [12]. Koopmans’ approximation for the determination of global descriptors such as chemical softness, hardness, electrophilicity index, ionization potential and electron affinity were also employed. However, the HOMO–LUMO and band gap values were obtained by lodging the formatted checkpoint file of the titled structure into Multiwfn software [13]. Energy optimization for the calculation of Natural Bond Orbital (NBO), Nonlinear optics (NLO), Natural Population Analysis (NPA) and Mulliken population analysis (MPA) by DFT method at CAM-B3LYP/6–31 + G(d) basis set was also carried out through the aid of Gaussian 09 W and GaussView 6.0 software. More so, Time Dependent-density Functional Theory (TD-DFT) method at B3LYP/6-31G + (d) basis set was used in computing UV–Visible spectroscopy. Proton nuclear magnetic resonance (^1^HNMR) was calculated using the Gauge-invariant atomic orbital (GIAO) and DFT at B3LYP/6-31G + (d) basis set. The Fourier transform infrared (FT-IR) was computed using Gaussian 09 W and GaussView 6.0 software while the analysis was done using vibrational energy distribution analysis (VEDA 4) programme [14] on the basis of their potential energy distribution (PED) assignments. ADCH, CHELPG and Density of states (DOS) were computed with the aid of Multiwfn software. Also, chemical electrostatic potential map (ESP) was computed using Multiwfn and VMD software [15] to show graphical representation of electron density in the studied compound.

## Results and discussion

### Frontier molecular orbital (FMO) analysis

**Fig. 2:**
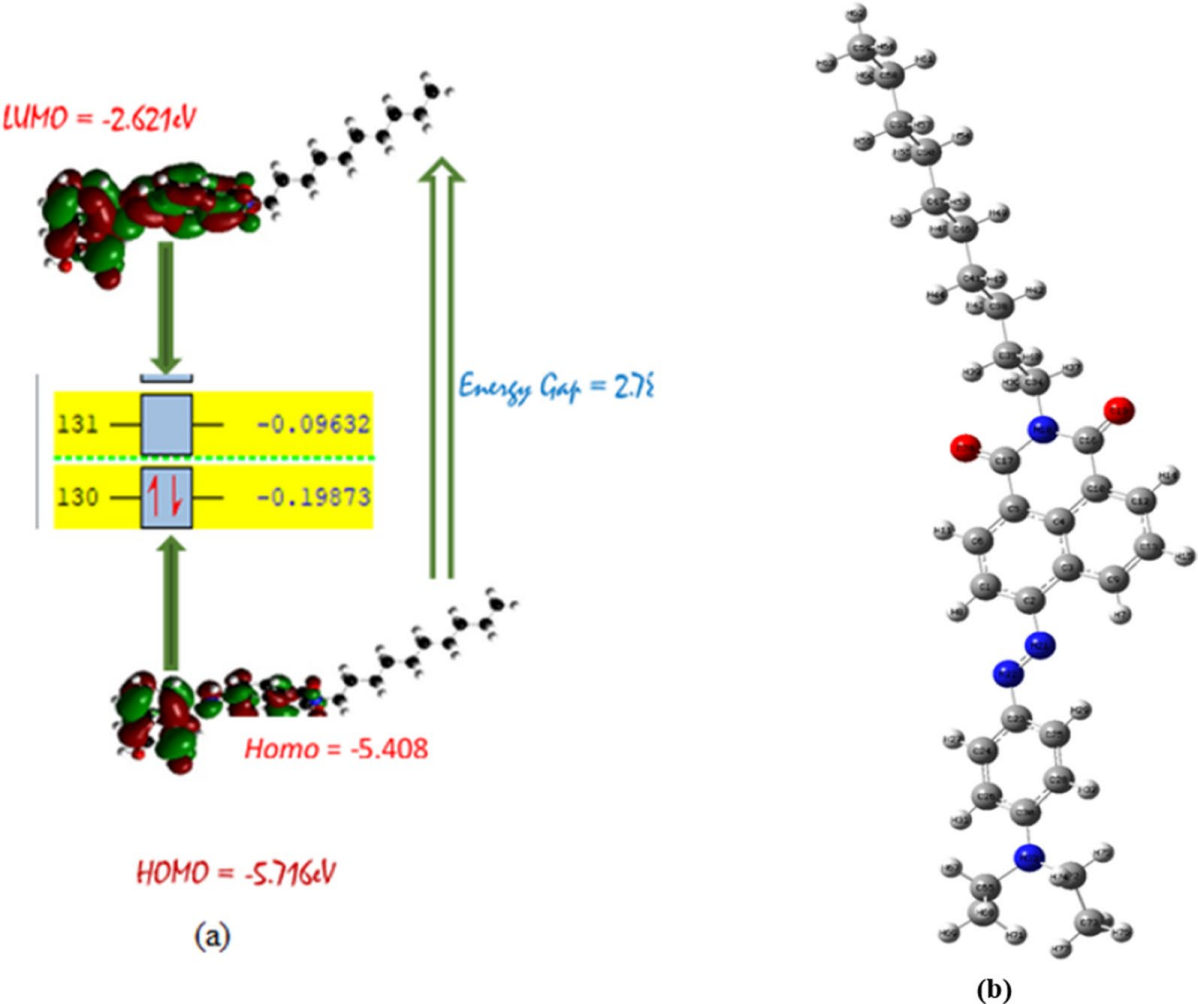
**a** HOMO-LUMU plot **b** Structural atomic label

The highest occupied molecular orbital (HOMO) and lowest unoccupied molecular orbital (LUMO) are parameters used in determining the chemical and electronic reactivity of compounds [16–19]. Figure 2a and b depicts the HOMO–LUMO, band (Energy) gap and the structural atomic label of the titled molecule. Frontier molecular orbitals energy is important in characterizing several parameters that affect the reactivity and stability of a molecule. All the global reactivity parameters of a molecule can be characterized using the FMOs. The energy gap is obtained from FMOs by subtracting the energy of the LUMO from the HOMO ($${E}_{HOMO}-{E}_{LUMO})$$ and these in turn are used to describe important properties such as stability, reactivity, hardness, and non-linear-optical properties of the molecule [19, 20]. The value of the energy gap will describe the light harvesting efficiency of the molecule. A smaller band gap indicates strong molecular interaction and therefore inter fragment charge transfer (ICT) ICT within the molecule. This in turn is used to predict the increase in solvent polarity and the tendency to accelerating rate of reactions. The isosurface of the $${E}_{HOMO}$$ and $${E}_{LUMO}$$ for the ADCP modeled structure were calculated by DFT/B3LYP method with 6-31G + (d) basis set.

From Fig. 2a, it can be seen that the density of the HOMO is localized on the naphthalene ring and partly on the benzoisoquinolinedione ring with a value of − 5.408 eV. However, the LUMO electron density is seen to be localized totally on all the aromatic ring atoms including the N-atoms with a value of − 2.621 eV. Further investigations made as to constantly explain the specific orbitals in which the HOMO and LUMO resides showed that HOMO (orbital 139), is on N22, C54, C55, C59, C61, C64 and O69 while the LUMO (orbital 140) resides on C2, C6, C17, C55, O19, N21 and N22 atoms respectively. This suggests a donor–acceptor electron movement after energy absorption [21]. Furthermore, the HOMO–LUMO energy gap which corresponds to the difference between HOMO and LUMO energies of the studied compound is theoretically found to be at a value of 2.806 eV (Table 1) which indicates the compound’s stiff and smooth nature. This accounts for the less stability and high chemical reactivity of the compound [22, 23].

**Table 1:** **a** HOMO–LUMO energy gap. **b** Chemical quantum descriptors

a			
Phase	HOMO	LUMO	Energy gap
Gas	− 5.4082	− 2.6213	2.7869
DMSO	− 5.3731	− 2.8109	2.5622
Ethanol	− 5.3748	− 2.8060	2.5688
Water	− 5.3723	− 2.8134	2.5589

b							
Phases	IP	EA	µ	η	Χ	ω	Σ
Gas	5.4082	2.6213	4.013	2.7869	− 4.013	2.8919	0.179
DMSO	5.3748	2.8109	4.092	2.5622	− 4.092	3.2676	0.195
Ethanol	5.3748	2.8060	4.090	1.2844	− 4.090	6.5133	0.389
Water	5.3723	2.8134	4.093	1.2795	− 4.093	6.5465	0.390

### EHOMO-ELUMO analysis and other quantum descriptors

Koopman’s approximation was used in computing global descriptors such as ionization energy (I) and electron affinity (A) given as;

1 $${\text{I }} = \, - {\varepsilon}_{{{\text{HOMO}}}}$$

2 $${\text{A }} = \, - {\varepsilon}_{{{\text{LUMO}}}}$$

Other global descriptors calculated are electronegativity (χ), electrochemical potential ($\mathop \upsilon \limits^{\prime }$), hardness (η), softness (S) and electrophilicity index (ω) expressed as;s

3 $$\chi = - \mathop \upsilon \limits^{\prime } = \frac{{{\text{I}} + {\text{A }}}}{2}$$

4 $$\upeta =\frac{\mathrm{I}-\mathrm{A }}{2}$$

5 $$\mathrm{S}=\frac{1 }{2\upeta }$$

6 $$\upomega =\frac{\upmu 2 }{2\upeta }$$

Electrophilicity index is the extent of stability experienced by a system when additional charges from the surroundings flow into the system [24, 25]. Hence, the electrophilicity index of the studied compound with a value of 5.939 eV indicated a high flow of electrons during donor–acceptor interaction. More so, the chemical hardness agrees with those reported in literature and is directly connected with the high reactivity and low stability of the compound [26]. The electrochemical potential of the compound is a determinant of the outflow of electrons from the molecule. The HOMO–LUMO energy gap shows the effect of different solvents on the polarity of the phases. Phases with smaller energy gap are more polar. It is worth knowing that an increase in solvent polarity accelerates the rates of reactions as a result, polarity plays a key role in solubility [27]. From the results obtained, we observed that the polarity follows a decreasing order as water > DMSO > Ethanol > Gas. The high polarity of water is due to it geometry, this is because of the bent shape of the molecules, the hydrogen and the Oxygen are both acting as nonmetals, under ordinary conditions, but the oxygen being more electronegative than hydrogen, so the two atoms form a covalent chemical bond making it more polar as compared to the other studied phases.

Imperatively, from the investigated descriptors (Table 1), it was observed that the solvent DMSO is more electronegative and as such, the more its tendency to attract electrons to itself. While water has the least electronegativity and as such it has the more tendency to donate electrons [28].

### Spectral analysis

#### Vibrational analysis (FTIR)

The studied compound was observed to have a 3n-6 modes of vibrations (n represents the number of atoms, with n = 72 atoms) [29, 30]. The compound has 210 vibrational modes out of which 71 were stretching (49 symmetric and 22 asymmetric stretching), 70 in-plane bending 56 torsional (37 symmetric and 19 asymmetric tor) and 13 out of plane bending (7 symmetric and 6 asymmetric out of plane) vibrations. Comparison of the experimental and theoretical results with the potential energy distribution (PED) assignments of the studied compound is presented in Table 2. Furthermore, optimization of the benzeneisoquinolinedione was achieved by Density Functional Theory (DFT) method at B3LYP/6-31G (d) basis set using Gaussian09W and GaussView6.0.16 softwares [9] while the spectrum (Additional file 1: Fig S2) was plotted using Multiwfn software [13]. A scale factor of 0.977 was employed in the spectrum plotting since it is the most recommended value for B3LYP/6-31G (d) functional.

**Table 2:** Experimental and theoretical vibrational energy distributional analysis of the studied compound (benzoisoquinolinedione)

Experimental wave number (cm^−1^) Unscaled	Theoretical wave number (cm^−1^) Scaled	PED Assignment (%)	Raman Activity
3375	3625	υOH (100)	2.2304
3063	3067	AsyυCH (82)	2.6607
2921	2940	AsyυCH (91)	7.5811
1699	1697	AsyυOC (83)	4.9258
1658	1648	AsyυCC(52) υCC(11)	7.0624
1618	1624	AsyυCC (45)	15.2705
1588	1576	βHOC (19)	34.0269
1382	1391	υCC (40)	38.9449

### C – H vibrations

C–H stretching vibrations are reported to be at 3100–3000 cm^−1^ [31–33]. C–H asymmetric stretching vibration which corresponds to the CH_3_ group on the alkyl chain of the compound under study was experimentally observed at 3063 cm^−1^ and 2921 cm^−1^ and theoretically calculated at 3067 cm^−1^ and 2940 cm^−1^ with a PED contribution of 82% and 91% respectively. (Table 2 and (Additional file 1: fig. S1, S2).

### O–H vibrations

Within the IR region of 3650 cm-^1^ to 3600 cm-^1^, free O–H stretch is accompanied with a sharp absorption peak. When the alcohol is dissolved in a solvent, this appears as a hydrogen bound O–H peak [34]. Table 2 clearly shows that the O–H stretching vibration of benzoisoquinolinedione was experimentally observed to have a wave number of 3375 cm-^1^ and theoretically calculated to have a wave number of 3625 cm-^1^ with a PED contribution of 100%.

### *C*=*C vibrations*

Aromatic rings C=C typically appear between 1600 cm-^1^ and 1450 cm-^1^, with poor overtone bands at 2000–1667 cm-^1^ used for aromatic ring substitution assignment [35]. At 1699, 1658, and 1618 cm-^1^, the aromatic C=C experimental stretching vibration of benzoisoquinolinedione was observed. However, theoretical calculated values of 1695, 1648, and 1624 cm^−1^ were found, with PED assignments of 65%, 52%, and 45%, respectively. This could be owing to the conjugation effect, which enhances the single bond character of the C=O and C=C bonds in the resonance hybrid, causing the force constant and related frequency to decrease. Furthermore, the stretching vibrations recorded empirically at 1382 cm-^1^ and theoretically calculated at 1391 cm-^1^ with a PED contribution of 40% might be attributed to C–C single bond symmetric stretching vibrations.

### C–O–H vibrations

C–O–H bending vibrations are typically observed as a weak and broad absorption band in the 1440–1220 cm^−1^ region [36]. The experimental C–O–H bending vibration of the examined compound was observed at 1382 cm^−1^ and theoretically computed at 1391 cm^−1^ with a PED contribution of 19%. The FT-IR data obtained for both experimental and theoretical investigations are found to be in perfect agreement, which is due to the theoretical study being a perfect confirmation of the previous experimental work because it also aligns with published research. Table 2 of the supporting documentation contains information on the geometrical properties of the compound.

### ^***1***^***HNMR analysis***

The reference solvent for the experimental ^1^HNMR examination of benzoisoquinolinedione was deuterium chloroform (CDCl3). The theoretical ^1^HNMR was performed in chloroform as the solvent, with the B3LYP/6-31G (d) basis set using the Gauge–invariant atomic orbital (GIAO) method [37] and tetramethylsilane (TMS HF/6-31G(d) GIAO) as the internal standard. Table 3 shows the experimental and theoretical ^1^HNMR chemical shift values in parts per million (ppm) as well as the corresponding assignments. The observed and theoretical chemical shift values of 0.88 and 0.85 ppm, respectively, indicate the presence of -CH_3_ protons (H_53_) at some distance from electronegative atoms in benzoisoquinolinedione. The observed and theoretical chemical shift values calculated at 1.26–1.74 ppm and 1.34–1.76 ppm (triplet, H_46_–H_29_) in the examined molecule show -CH_2_ protons of the alkyl chain. The experimental and theoretical chemical shift values of several multiplets found at 4.03 and 4.07 ppm (H_25_) due to -NH protons of aromatic rings in benzoisoquinolinedione were very fascinating. The presence of –CH_2_ protons directly linked to the aromatic ring was detected at 6.52 ppm and 6.39 ppm (H_67_) for the experimental and predicted values, respectively (Table 3 and Fig. S3 and fig. S4). The observed theoretical chemical shift values of H_65_, H_68_, H_15_, H_60_, H_8_ and H_62_ at 7.20, 7.45, 7.64, 7.98, 8.19 and 8.49 ppm correspond perfectly with the experimental chemical shift values of 7.26–7.41, 7.49, 7.65, 7.99, 8.20, and 8.40 ppm, indicating aromatic ring -CH protons. The experimental chemical shift value of 16.67 ppm, however, could not be theoretically verified. This could be ascribed to the proton (shigh)’s de-shielding level and very downfield position in the said region. It should be emphasized that the primary protons responsible for the observed chemical shift values are those of -CH_3_, − CH_2_, − NH, and − CH of the aromatic rings of the molecule under consideration.

**Table 3:** Comparison of the experimental and theoretical ^1^HNMR result of benzoisoquinolinedione

S/No.	Experimental (ppm)	Theoretical (ppm)	Assignment
1	0.88	0.85	Singlet, -CH_3_ protons at some distance from electronegative atoms (H_53_)
2	1.26–1.74	1.34–1.76	Triplet, -CH_2_ protons of the alkyl chain (H_46_–H_29_)
3	4.03	4.07	Multiplet, -NH protons of aromatic ring (H_25_)
4	6.52	6.39	Triplet, -CH protons of aromatic ring (H_67_)
5	7.26–7.41	7.20	Duplet, -CH protons of aromatic ring (H_65_)
6	7.49	7.45	Multiplet, -CH protons of aromatic ring (H_68_)
7	7.65	7.64	Duplet, -CH protons of aromatic ring (H_15_)
8	7.99	7.98	Multiplet, -CH protons of aromatic ring (H_60_)
9	8.20	8.19	Duplet, -CH protons of aromatic ring (H_8_)
10	8.40	8.49	Triplet, -CH protons of aromatic ring (H_62_)
11	16.67	–	--------

### UV–VIS spectroscopy

**Fig. 3:**
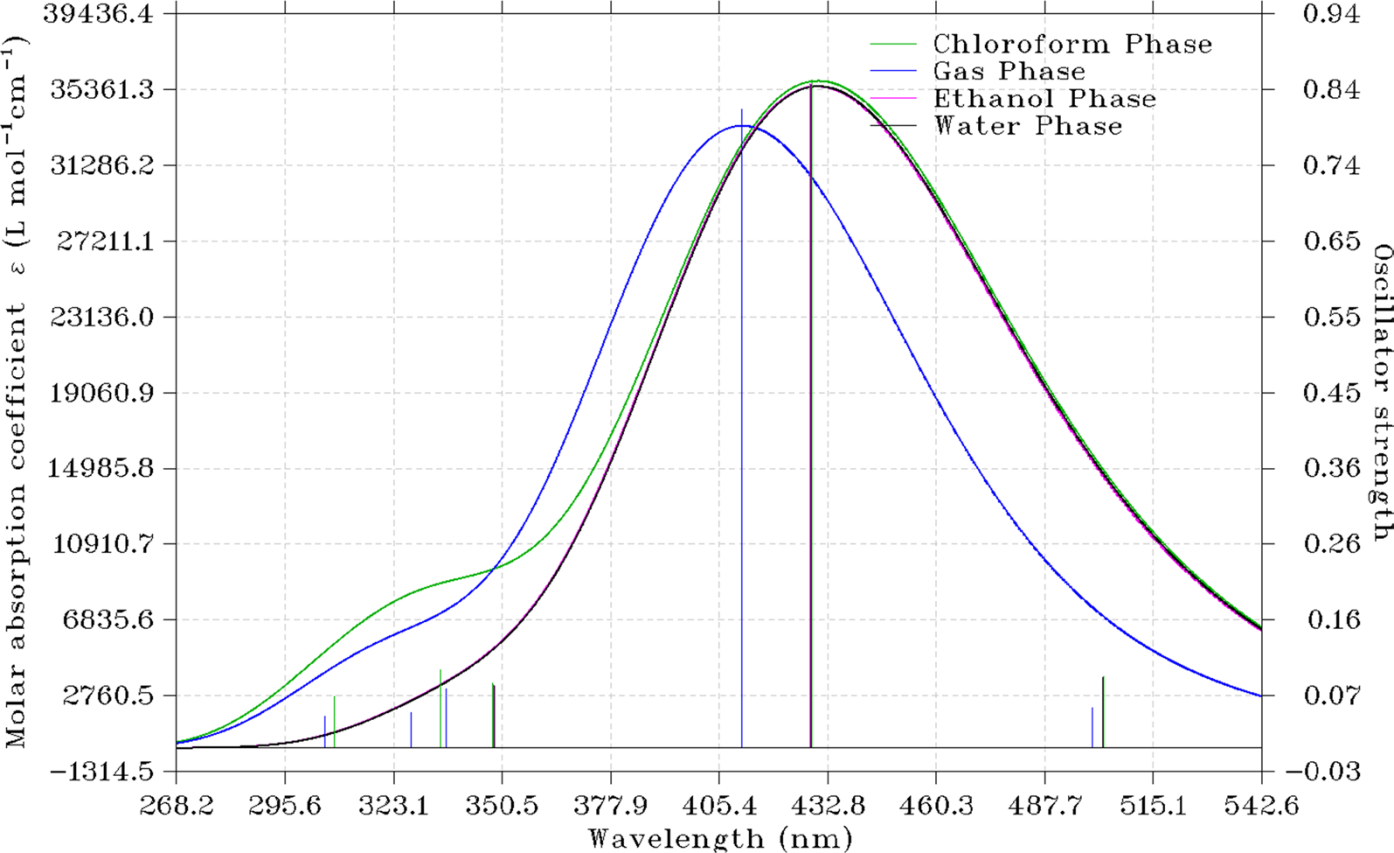
UV–Visible spectrum of the different phases of the studied compound

The basis for electronic spectra calculation is dependent on the vast chemical and physical molecular properties. By modifying the spectra characteristics of molecules, several physical chemical and effects can be computationally investigated as most chemical properties of molecules are embedded in both ground and excited states of the molecule [18, 38]. The electronic activities of the titled compound in gas phase and different solvents such as DMSO, ethanol and water was estimated by DFT/B3LYP/6–311 + G(d,p). The wavelength for the vertical excitation observed from the studied compounds and the changes in the wave length have effects on the spectral line. The absorption bands in the region 290–300 nm are attributed to n → π* transitions as shown in Table 4 and Fig. 3 in comparison with the experiment excitation energies. From this analysis, vertical excitation of DMSO is quite different from gas and other solvent under-study due to solvatochromic effects which is due to changes in the dielectric constant of the solvent.

**Table 4:** UV Transition Analysis for the Vertical Excitation experimental and theoretical

Phases	Excitation type	Energy/eV theory	Energy/eV experiment	Λ(nm)	F	Major contributions (%)
Gas	S_0_ → S_1_	2.6172	2. 6472	473.73	0.0047	125 → 131(78.63)
						128 → 132(15.89)
DMSO	S_0_ → S_1_	2.3293	2.3579	532.28	1.0154	129 → 131(46.60)
						130 → 131(88.33)
Ethanol	S_0_ → S_1_	2.6295	2.7608	471.51	0.0347	128 → 131(74.33)
						130 → 131(2.36)
Water	S_0_ → S_1_	2.6308	2.6816	471.29	0.0344	128 → 131(74.27)
						128 → 132(18.04)

### Fragment density of states (DOS)

**Fig. 4:**
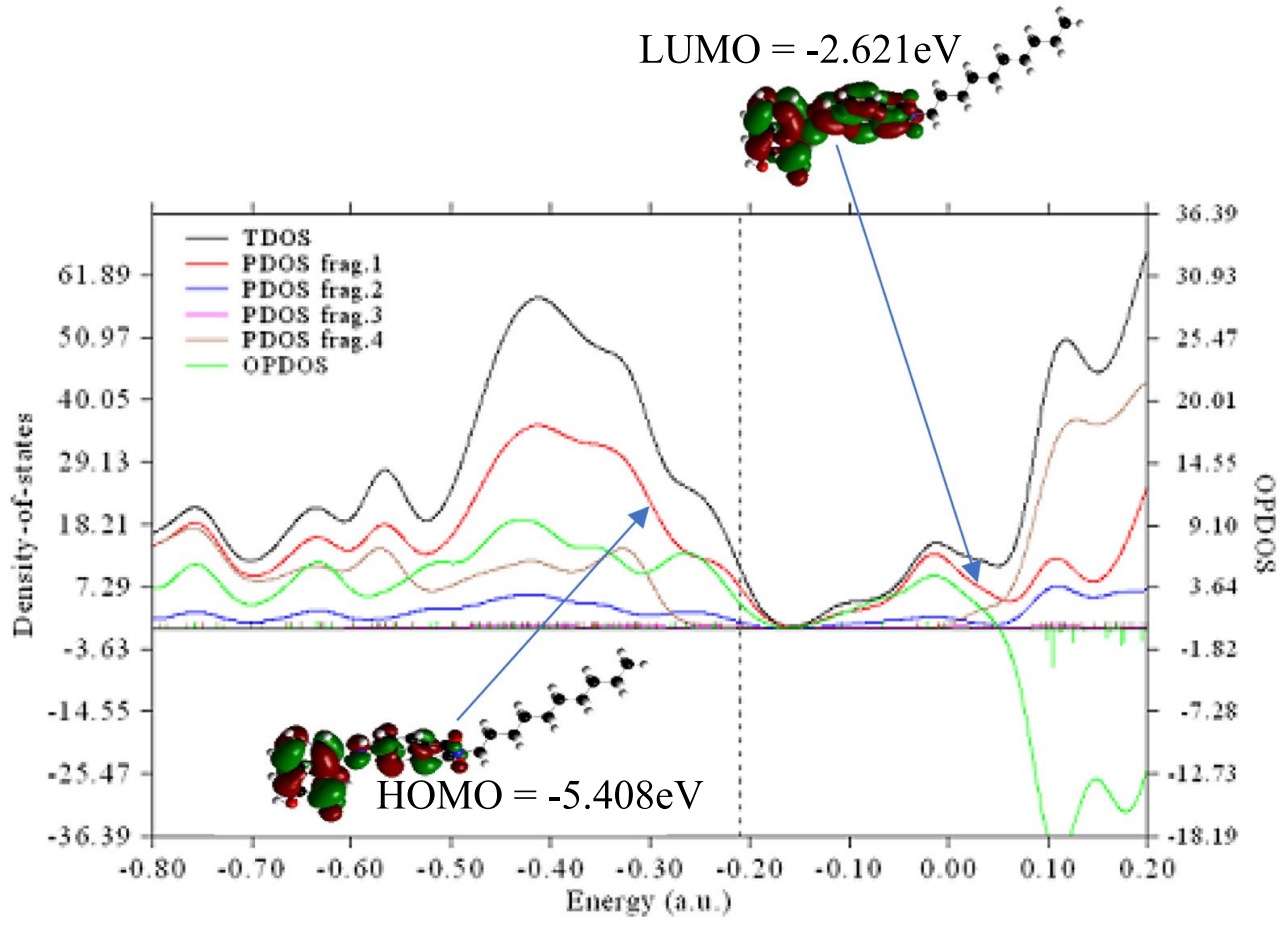
Density of states of the studied compound computed using Multiwfn software

Total Density of State (TDOS), Overlap partial density of state (OPDOS) and partial density of state (PDOS) were calculated to explain its electronic characteristics [39, 40]. However, the graphs generated by DOS are critical tools for analyzing the nature and structure of electrons. The PDOS and OPDOS curves are useful in visualizing orbital composition [41]. To determine the degrees of contribution of each fragment, the benzoisoquinolinedione is divided into four atomic fragments. Fragments 1, 2, 3, and 4 indicate the contributions of carbon, hydrogen, oxygen, and nitrogen atoms, and are represented by (red, blue, magenta, and purple) colours, as illustrated in Fig. 4.

The discrete vertical lines signify molecular orbitals and the dashed lines represent the highest occupied molecular orbitals (HOMO). From the graph, the highest contribution was made by carbon (fragment 1) with red curve as seen in the HOMO. Howbeit, the positive OPDOS value between 3.64 and 9.10 a.u (green curve), which corresponds to bonding between fragments 1 and 4 (red and purple curve) suggests that carbon is important for the stabilization of nitrogen atoms. Fragment 3, (magenta curve) gave little or no contribution to the HOMO–LUMO. This could be attributed to the -I effects of oxygen atom though its contribution was sparingly made to the molecular orbital between -0.50 to -0.30 a.u. Moreso, the negative value of OPDOS at -1.82 to -18.19 a.u region implies antibonding characteristics between fragment 1 and 2 [42] which is due to the unfavourable overlapping in the orbital phase. It could therefore be concluded that the major contributions made by carbon and nitrogen atoms to the HOMO–LUMO was as a result of the clouded electron density on carbon and nitrogen the major contribution depict the structural attribute to the light harvesting efficiency and the photovoltaic properties of the studied compound.

### Atomic charge analysis

Atomic charge is one of the most important concepts in chemistry. It provides a simple picture of electron density distribution within a molecule [43]. Atomic charges are very important in understanding of structure–property relation of molecules. There are various ways to calculate atomic charges and they serve different purposes. The atomic charges of the target molecule as shown in Additional file 1: Table S1 of the supporting information were obtained by Atomic Dipole Moment Corrected Hirshfeld Charges (ADCH) from electrostatic potentials using a Grid Based method (CHELPG), Mulliken Population Analysis (MPA) and Natural Population Analysis (NPA) methods. The NPA and MPA are basis set sensitive, charge in basis sets bring about change in calculated net charges. However, NPA gives a better charge distribution since its calculation is based on the natural charge [44]. CHELPG charges are fitted to reproduce charges at a number of points around the molecule [45] and so are not very suitable for the treatment of large systems where some of the innermost atoms are located far away from the points at which the molecular electrostatic potential is computed. Charges predicted from ADCH are very efficient and insensitive to basis set. However, the predicted charges are usually smaller than that of Mulliken. ADCH and CHELPG charges were computed using Multiwfn analyzer while NPA and MPA charges were computed from Gaussian 09 W and GaussView 06 [9] software. Comparing the charges obtained using the various charge population methods listed above, the results showed that charge values for all the heteroatoms (oxygen and nitrogen) are negative for all the methods of calculations used in the sequence ADCH < CHELPG < MPA < NPA with the negative charge value of oxygen higher than nitrogen. This observation may be due to the high electronegativity of oxygen compared to nitrogen. Also, the charge values for all the hydrogen atoms are positive across all the population methods used except for some negligible few exceptions in CHELPG values of H_28_, H_29_, H_31_, H_32_, H_33_, H_34_, H_37_, H_38_, H_40_, H_41_, H_43_, H_45_, H_46_, H_49_ and H_50_ where, the charge values are slightly negative. The positive charge values on hydrogen may be due to the fact that the atoms surrounding hydrogen in the studied compound are higher in electronegative values than hydrogen. Also, the slight negative charge on the above listed hydrogen atoms may be attributed to the electron donating effect of the two hydroxy groups attached to the naphthalene ring. The carbon atoms in the benzoisoquinolinedione base on their positions have diverse positive and negative charges for all the population analysis methods used with carbon 16 (C_16_) having the highest positive charge of 0.71306 e and carbon 48 (C_48_) having the highest negative charge of − 0.68015 e, both from NPA charges. The overall result is in agreement with similar work in the literature [24].

### Non-linear optics (NLO)

The total statics dipole moment (µ), the mean polarizability (α), the anisotropy of the polarizability (∆α) and the first hyperpolarizability (β) using x, y, z components were calculated because of its importance in providing key functions of frequency shifting, optical modulation switching, laser, fiber, optical emerging technologies in region such as telecommunications, signal processing and optical interconnections as reported by Wada, O. (2004) [46]. The calculated values are presented in Table 5. The magnitude of the molecular hyperpolarizability β is one of the key functions in NLO system. The analysis of the β show an increase with polarity. Increase in solvent polarity brings about the increase in hyperpolarizability. NLO parameters were calculated using Eqs. (7–10).

7 $$\mu =\sqrt{({\mu }_{x}^{2}+ {\mu }_{y}^{2}+ {\mu }_{z}^{2})}$$

8 $${\mathrm{\alpha }}_{\mathrm{total}}= \frac{({\propto }_{xx}+{\propto }_{yy}+{\propto }_{zz})}{3}$$

9 $${\Delta }_{\mathrm{\alpha }}= \sqrt{(({\propto }_{xx}-{\propto }_{yy}{)}^{2}+ ({\propto }_{yy}-{\propto }_{zz}{)}^{2}+({\propto }_{zz}-{\propto }_{xx}{)}^{2})}$$

10 $$\beta_{{{\text{total}}}} = \sqrt {(\beta_{{\text{x}}}^{2} + \beta_{{\text{y}}}^{2} + \beta_{{\text{z}}}^{2} )}$$

where, $\mu$ is the total dipole moment along x, y, and z axis.

**Table 5:** Non – Linear Optics (NLO)

Phases	µ_total_	α_total_	∆µ_total_	β_total_
Gas	4.1434	463.46	17228.09	804.26
DMSO	5.5748	649.773	1124.86	69314.86
Ethanol	5.5341	644.0527	1113.42	66816.769
Water	5.5936	652.427	1129.78	10297.57

${\mathrm{\alpha }}_{\mathrm{total}}$, the mean polarizability or total polarizability.

${\Delta }_{\mathrm{\alpha }}$, the anisotropy of the polarizability and, ${\upbeta }_{\mathrm{total}}$, the mean first hyperpolarizability. The dipole moment observed along the z-axis (μ_z_ = 0.503D) was found to be the highest. In this study, the values of ${\mathrm{\alpha }}_{\mathrm{total}}$, ${\Delta }_{\mathrm{\alpha }}$ and ${\upbeta }_{\mathrm{total}}$ were converted from atomic units (a.u) to electronic units (esu) ($\mathrm{\alpha },$ 1a.u = 0.1482 × 10^–24^ esu and for $\upbeta ,$ 1a.u = 8.6393 × 10^–33^ esu). The dipole moment and mean first hyperpolarizability values of the studied compound were theoretically calculated at 2.153 D and 7.289 × 10^–30^ esu, respectively as against the reference (DMSO), which was computed to be 1.3732 D and 0.3728 × 10^–30^ esu. Also observed were the values of the mean polarizability or total polarizability and the anisotropy of the polarizability at 6.412 × 10^–23^ and 4.851 × 10^−23^esu, respectively. This result implies that the total dipole moment of the studied compound is 1.57 times greater than the reference. Also, the value of 7.289 × 10^–30^ esu for DMSO shows that the studied compound is 19.6 times greater than the value of the gas solvent signifying a better nonlinear optical activity in the studied compound than the reference and could be used in optoelectronic devices. This is in agreement with the frontier molecular orbital analysis, such that from the quantum descriptors calculated in different solvents DMSO is more electronegative and as such, the more its tendency to attract electrons to itself. While water has the least electronegativity and as such it has the more tendency to donate electrons.

### Natural bond orbital (NBO) analysis

Natural bond orbital (NBO) analysis is one of the many available options for translating computational solutions of Schrodinger’s wave equation into the familiar language of chemical bonding concepts [47]. It is used to determine resonance structure contributions to molecules and hence through the help of bonding and antibonding orbital interactions provides an efficient method to study intramolecular and intermolecular charge transfer interactions and delocalization of electron density within the molecules [48]. NBO analysis for this study was performed using the Gaussian 09 W [9] and GaussView 06 software with the DFT method at B3LYP/ 6-31G(d) levels. The strength of delocalization interactions (stabilization energy) for each donor NBO (i), acceptor NBO (j) and E^(2)^ associated with electron delocalization between the donor and the acceptor is predicted by the second order energy [49].

11 $${E}^{(2)}={n}_{r}\frac{{(F(i,j))}^{2}}{E\left(j\right)-E(i)}$$

where n_r_ is the population of the donor orbital, Fij is the off-diagonal Fock–matrix element, E(j)–E(i) is the difference in orbital energies of the donor and the acceptor NBO orbitals. Summary of the NBO output result analysis showed a total Lewis structure of (97.45%), Core (99.96%), Valence Lewis (96.53%), non-Lewis’s structure (2.5%), Valence Non-Lewis (2.32%) and Rydberg Non-Lewis (0.18%) in the studied compound. Additional file 1: Table S1 of the supporting information show the calculated occupancies of natural orbitals (Lewis and Non-Lewis type σ and π—bonding orbitals). From the tables, it is observed that π(N21 – N22) bond have the lowest occupancy of 0.1963 e which is formed from a hybrid of SP^99.99^ on nitrogen 22 (99.58%, P character) interacting with π(C54–C56) formed from a hybrid of sp^1.00^ (99.98% p- character). π(C9–C13) bond with occupancy of 0.2350 e which is formed from a hybrid of sp^1.00^ on Carbon 13 (99.94% p-character) is observed to interact with π(C10–C12) forming a hybrid of sp^1.00^ with 99.98% p-character. The bond between σ(C66–O71), has the highest occupancy of 1.99496 e formed from a hybrid of sp^3.12^ on oxygen 71 (75.57% p-character) interacting with lone-pair, LP(1)N_18_ with SP^99.99^ hybrid (99.95% P-character. Also, σ(C17–N18) show a high occupancy of 1.99440 e formed from a hybrid of SP^2.09^ on nitrogen 18 (67.57% P-character).

The Natural Bond orbital analysis was also studied at the DFT/B3LYP/-311 + G(d,p) level using NBO 3.1 program as performed in the Gaussian 09 W software package with the aim to qualitatively measure the intermolecular interaction of the electrons in the studied phases. The main donor–acceptor orbitals in the studied solvents was observed from the anti-bonding orbitals π*C_26_-C_30_ to the anti-bonding of the π*C_25_-C_28._ The highest stabilization energy was observed from the gas while the least stabilization energy observed from ethanol phase with 178.77 kcal/mol as shown in Table 6.

**Table 6:** Second order perturbation energy for benzoisoquinolinedione

Phases	Donor	Acceptor	${{\varvec{E}}}^{(2)}$	E(j) -E(i)	F(ij)
Gas	π*C_23_-C_30_	π*C_23_-C_24_	267.12	0.01	0.082
DMSO	π*C_26_-C_30_	π*C_25_-C_28_	178.52	0.02	0.078
ETHANOL	π*C_26_-C_30_	π*C_25_-C_28_	177.99	0.02	0.078
WATER	π*C_26_-C_30_	π*C_25_-C_28_	178.77	0.02	0.078

A second order perturbation theory analysis was also carried out to investigate potential interactions between all bonding NBOs and the non-Lewis or acceptors. As shown in Additional file 1: Table S1, for each donor (i), the higher the perturbation energy value, the stronger the interactions between electron donors and acceptors, and the more intensely conjugated the system [40]. Table 6 shows the most significant intramolecular hyperconjugative interactions that result in the highest stabilization energy detected in the examined molecule. These significant hyperconjugative interactions and the value of their stabilization energies observed for NBO analysis of benzoisoquinolinedione were π(C55–C58) → π(C54–C56) 316.15 kcal/mol, π(C3–C4) → π(C1–C2) 309.08 kcal/mol, π(C63–C66) → π(C57–C61) 242.54 kcal/mol, π(C17–O20) → π(C5–C6) 155.87 kcal/mol, π(C16–O19) → π(C10–C12) 124.94 kcal/mol, LP(1) N18 → π(C17–O20) 67.03 kcal/mol, π(N21–N22) → π(C54–C56) 41.03 kcal/mol, LP(2) O69 → π(C57–C61) 37.46 kcal/mol, LP(2) O71 → π(C63–C66) 32.47 kcal/mol and π(C9–C13) → π(C10–C12) 29.68 kcal/mol. These strong interactions within the ring system as observed in the results suggest an intense delocalized structure and the extra stability for benzoisoquinolinedione can be attributed to resonance stabilization. This strong interaction was also observed in the higher LHE of the studied compound in the different solvents respectively.

### Photovoltaic properties

The energy difference between the redox potential of the electrolyte's redox couple $\left({I}^{-}/{I}_{3}^{-}\right)$ and the quasi-Fermi level of the semiconductor's conduction band $\left({TiO}_{2}\right)$ is defined as the open circuit voltage (${V}_{OC}$). It is expressed mathematically using an Eq. (12).

12 $${V}_{OC}=\frac{{E}_{CB}+\Delta CB}{q}+\frac{kT}{q}In\left(\frac{{n}_{c}}{{N}_{CB}}\right)-\frac{{E}_{Redox}}{q}$$

where ${E}_{CB}$ denotes the ${TiO}_{2}$ conduction band edge, q the unit charge, T the absolute temperature, k the Boltzmann constant, ${n}_{c}$ the number of electrons in the conduction band, ${N}_{CB}$ the density of accessible states in the conduction band, and ${E}_{redox}$ the electrolyte's redox potential. $\Delta$ CB is the shift in CB caused by dye adsorption. Where it is represented numerically by an Eq. (13).

13 $$\Delta CB=\frac{q{\mu }_{normal}\gamma }{{\varepsilon }_{0}\varepsilon }$$

where ${\mu }_{normal}$ is the dipole moment of the individual solvent molecule perpendicular to the surface of ${TiO}_{2}$, and $\gamma$ is the solvents surface concentration, ${\varepsilon }_{0}$ and $\varepsilon$ are the vacuum permittivity and dielectric permittivity respectively. The calculation of ${V}_{OC}$ can also be approximately obtained by the difference between ${E}_{LUMO}$ and ${E}_{CB}$. However, it is used for this purpose because the studied dye is singly not in the adsorbed state on ${TiO}_{2}$. Therefore, calculations of ${n}_{c}$ and ${N}_{CB}$.

${J}_{sc}$ can be mathematically calculated using Eq. (14).

14 $${J}_{SC}=\int LHE(\lambda ){\phi }_{inject}{\eta }_{collect}d\lambda$$

LHE (λ) is the light harvesting efficiency at maximum wavelength, ${\phi }_{inject}$ is the electron injection efficiency, and ${\eta }_{collect}$ is the charge collection efficiency. To obtain a high ${J}_{SC}$, LHE and ${\phi }_{inject}$ should be as high as possible. The LHE can be mathematically expressed using Eq. (15).

15 $$LHE=1-{10}^{-f}$$

where f is the oscillator strength of the solvents corresponding to ${\lambda }_{max}$, ${\phi }_{inject}$ is related to the thermodynamic driving force ${\Delta G}_{inject}$ of electron injection from the excited states of solvent to conductive band ${TiO}_{2}$

${\Delta G}_{inject}$ (The free energy difference for electron injection) is mathematically represented with the aid of Eq. (16).

16 $${\Delta G}_{inject}={E}^{dye*}-{E}_{CB}^{{TiO}_{2}}\approx {E}^{dye}+\Delta E-{E}_{CB}^{{TiO}_{2}}$$

where ${E}^{dye*}$ is the redox potential of the oxidized dye at excited state. ${E}^{dye}$ is the redox potential of the oxidized dye at ground state and $\Delta E$ is the lowest vertical excitation energy. ${E}_{CB}^{{TiO}_{2}}$ is the energy of the conductive band edge of ${TiO}_{2}$.

$\Delta {G}_{reg}$ (The driving force for dye regeneration) is mathematically represented as:

17 $$\Delta G_{reg} = { }\phi \left( {I^{ - } /I_{3}^{ - } } \right) - E^{dye}$$

A value of ${\Delta G}_{reg}$ greater than 0.2 eV for an oxidized solvent could be the efficient electron injection [35]. In order to determine the value of ${J}_{SC}$ and the overall conversion efficiency (φ), the calculated values of ${V}_{OC}$,$f$,$LHE$, ${\lambda }_{max}$,${\Delta G}_{inj}$,${\Delta G}_{reg}$,${\Delta G}_{cr}$ in four different solvents along with the changes in wavelength are reported in Table 7. It should be noted that a solvent with a small energy band gap is beneficial to a red-shifted absorption spectrum and gives rise to more electrons corresponding to an increase in ${n}_{c}$ and thus, increases the efficiency of ${V}_{OC}$. In calculating ${\Delta G}_{reg}$, it is important to note that the experimental value of ${E}_{CB}$ used for the TiO_2_ semiconductor is − 4.03 eV [50]. It is observed that DMSO solvents ${\Delta G}_{inject}$ is greater than 6.8109 eV and therefore, all the dyes in the four phases provide efficient electron injection, However, the value of ${\Delta G}_{inject}$ for the solvents are in this order DMSO > ethanol > gas > water and DMSO, which is greatest in all the four phases provides the highest electron injection of all the LHE. It is also observed that the ${\Delta G}_{reg}$ for all the solvents in the four phases is less than 0.2 eV and hence, the solvent have low or no effect on ${\Delta G}_{reg}$. The results in Table 7 also shows that the circuit voltage gave a higher polarity with DMSO compared to other solvents in the four phases, the highest values of $f$ come from DMSO and ethanol but the value of LHE varies and shows greater stability in the following order: DMSO > ethanol > water, with DMSO having the highest value of ${V}_{OC}$ in all studied phases. Photovoltaic as the name implies is the best- known method for generating electric power by using solar cells to convert energy from the sun into a flow of electrons by the photovoltaic effect [51–54]. From the studied phases, DMSO was observed to have the highest light harvesting efficiency and this also correlated in its highest short circuit voltage making it the best solvent for this purpose compared to the other solvents.

**Table 7:** Photovoltaic properties of the four solvents in gas, DMSO, ethanol, and water phases

Phases	LHE	∆G^inj^	JSC	∆G_reg_	V_OC_	∆G_cr-_
Gas	0.0108	5.6097	0.0603	− 2.1787	− 1.379	− 8.8597
DMSO	0.9035	6.8109	6.1536	− 1.9891	− 1.189	− 10.061
Ethanol	0.0768	5.8032	0.4457	− 1.9940	− 1.194	− 10.056
Water	0.0760	5.8108	0.4420	− 1.9866	− 1.187	− 10.063

## Conclusion

The synthesis, characterization, spectral (FT-IR, NMR, UV) investigations, DFT studies, *and* investigation of a novel benzoisoquinoline azo compound as potential light harvesters has been carried out. The HOMO was localized on the naphthalene ring and partly on the benzoisoquinoline ring with a value of − 5.408 eV and the LUMO electron density was localized on all the aromatic ring atoms including the N-atoms with a value of − 2.621 eV. Comparing the charges obtained using the various charge population methods listed above, the results showed that charge values for all the heteroatoms (oxygen and nitrogen) are negative for all the methods of calculations used in the sequence ADCH < CHELPG < MPA < NPA, with the negative charge value of oxygen higher than nitrogen. This was due to the high electronegativity of oxygen compared to nitrogen. From the NBO analysis, it was observed that π(N21–N22) bond have the lowest occupancy of 0.1963 e which is formed from a hybrid of sp^99.99^ on nitrogen 22 (99.58%, p character) interacting with π(C54–C56) formed from a hybrid of sp^1.00^ (99.98% p-character). π(C9–C13) bond with occupancy of 0.2350 e which is formed from a hybrid of sp^1.00^ on Carbon 13 (99.94% p-character) was observed to interact with π(C10–C12) forming a hybrid of sp^1.00^ with 99.98% p-character. The bond between σ(C66–O71), has the highest occupancy of 1.99496 e formed from a hybrid of sp^3.12^ on oxygen 71 (75.57% p-character) interacting with LP (1) N18 with sp^99.99^ hybrid (99.95% p-character. The research results further showed an excellent agreement with other works, in which the benzoisoquinoline group acted as a charge acceptor in photo conversion process. Moreover, in the present study, shedding light on the photo-physics of the benzoisoquinolinedione molecule, will help to design new benzoisoquinolinedione-based compounds with improved light harvesting and light emitting properties.

## Supplementary Information

**Additional file 1: Table S1.** Different ADCH, CHELPG, MPA and NPA charges calculated for the studied compound. **Fig. S1.** Experimental FTIR Spectrum. **Fig. S2.** Theoretical FTIR Spectrum. **Fig S3.** Experimental 1HNMR Spectrum. **Fig. S4.** Theoretical 1HNMR Spectrum.

## Data Availability

All the analysis and other results were carried out at the center for high performance computer South Africa and more data for this study can be obtained from Hitler Louis at louismuzong@gmail.com.

## References

[1] Rostami-Tapeh-Esmail E, Golshan M, Salami-Kalajahi M, Roghani-Mamaqani H (2020). Perylene-3, 4, 9, 10-tetracarboxylic diimide and its derivatives: synthesis, properties and bioapplications. Dyes Pigm.

[2] Crespi S, Simeth NA, König B (2019). Heteroaryl azo dyes as molecular photoswitches. Nat Rev Chem.

[3] Freundlich JS, Wang F, Vilchèze C, Gulten G, Langley R, Schiehser GA, Jacobus DP, Jacobs Jr WR, Sacchettini JC (2009). Triclosan derivatives: towards potent inhibitors of drug-sensitive and drug-resistant Mycobacterium tuberculosis. ChemMedChem.

[4] Tahir T, Shahzad MI, Tabassum R, Rafiq M, Ashfaq M, Hassan M, Kotwica-Mojzych K, Mojzych M (2021). Diaryl azo derivatives as anti-diabetic and antimicrobial agents: synthesis, in vitro, kinetic and docking studies. J Enzyme Inhib Med Chem.

[5] Odey JO, Louis H, Agwupuye JA, Moshood YL, Bisong EA, Brown OI (2021). Experimental and theoretical studies of the electrochemical properties of mono azo dyes derived from 2-nitroso-1-naphthol, 1-nitroso-2-naphthol, and CI disperse yellow 56 commercial dye in dye-sensitized solar cell. J Mol Struct.

[6] Ghasemian M, Kakanejadifard A, Azarbani F, Zabardasti A, Kakanejadifard S (2014). Spectroscopy and solvatochromism studies along with antioxidant and antibacterial activities investigation of azo–azomethine compounds 2-(2-hydroxyphenylimino) methyl)-4-phenyldiazenyl) phenol. Spectrochim Acta Part A Mol Biomol Spectrosc.

[7] Romano E, Castillo MV, Pergomet JL, Zinczuk J, Brandán SA (2012). Synthesis and structural and vibrational analysis of (5, 7-dichloro-quinolin-8-yloxy) acetic acid. J Mol Struct.

[8] Babu NS, Vuai SA (2021). Theoretical studies of optoelectronic and photovoltaic properties of D-A polymer monomers by density functional theory (DFT). Des Monomers Polym.

[9] Yeşildağ A, Erdoğan M, Medetalibeyoğlu H, Horoz S (2022). Synthesis of benzidine-based conjugated organic materials bearing donor-acceptor groups: DFT studies and photovoltaic applications. J Mol Struct.

[10] Vuai SA, Khalfan MS, Babu NS (2021). DFT and TD-DFT studies for optoelectronic properties of coumarin based donor-π-acceptor (D-π-A) dyes: applications in dye-sensitized solar cells (DSSCS). Heliyon.

[11] Ameuru US, Yakubu MK, Bello KA, Nkeonye PO, Halimehjani AZ (2018). Synthesis of disperse dyes derived from 4-amino-N-decyl-1, 8-naphthalimide and their dyeing properties on polyester fabrics. Dyes Pigm.

[12] Li X, Li J, Gao Y, Kuang Yi, Shi J, Bing Xu (2010). Molecular nanofibers of olsalazine form supramolecular hydrogels for reductive release of an anti-inflammatory agent. J Am Chem Soc.

[13] Frisch, Michael J. "Gaussian09. 2009.

[14] Al-Aqar R (2020). A fluorescent molecule based on 1, 8-naphthalic anhydride: synthesis, spectral properties, and studying the conductance in solution. Egypt J Chem.

[15] Faggyas RJ, Sloan NL, Buijs N, Sutherland A (2019). Synthesis of structurally diverse benzotriazoles via rapid diazotization and intramolecular cyclization of 1, 2-aryldiamines. Eur J Org Chem.

[16] Enudi OC, Louis H, Edim MM, Agwupuye JA, Ekpen FO, Bisong EA, Utsu PM (2021). Understanding the aqueous chemistry of quinoline and the diazanaphthalenes: insight from DFT study. Heliyon.

[17] Lu T, Chen F (2012). Multiwfn: a multifunctional wavefunction analyzer. J Comput Chem.

[18] Nagayama T, Hirota T, Honma M, Kurayama T, Adachi Y, Tamura Y, Kanya Y (2020). VEDA: VERA data analysis software for VLBI phase-referencing astrometry. Publ Astron Soc Jpn.

[19] Humphrey W, Dalke A, Schulten K (1996). VMD: visual molecular dynamics. J Mol Graph.

[20] Adole VA, Jagdale BS, Pawar TB, Sawant AB (2020). Experimental and theoretical exploration on single crystal, structural, and quantum chemical parameters of (E)-7-(arylidene)-1, 2, 6, 7-tetrahydro-8 H-indeno [5, 4-b] furan-8-one derivatives: A comparative study. J Chin Chem Soc.

[21] Adole VA, Waghchaure RH, Pathade SS, Patil MR, Pawar TB, Jagdale BS (2020). Solvent-free grindstone synthesis of four new (E)-7-(arylidene)-indanones and their structural, spectroscopic and quantum chemical study: a comprehensive theoretical and experimental exploration. Mol Simul.

[22] Agwupuye JA, Louis H, Enudi OC, Unimuke TO, Edim MM (2021). Theoretical insight into electronic and molecular properties of halogenated (F, Cl, Br) and hetero-atom (N, O, S) doped cyclooctane. Mater Chem Phys.

[23] Ren Y, Li M-Y, Song Y-X, Sui M-Y, Sun G-Y, Xiao-Chun Qu, Xie P, Jing-Lan Lu (2021). Refined standards for simulating UV–vis absorption spectra of acceptors in organic solar cells by TD-DFT. J Photochem Photobiol A.

[24] Ameuru Umar Salami, Okah Peter Kayode, Saliu Hafsat Ronke, Yakubu Charles Itopa (2020). Synthesis and dyeing performance of azo-anthraquinone dyes on polyester fabrics. Sci Forum J Pure Appl Sci.

[25] Yee SK, Sun J, Pierre Darancet T, Tilley D, Majumdar A, Neaton JB, Segalman RA (2011). Inverse rectification in donor–acceptor molecular heterojunctions. ACS Nano.

[26] Zhang L, Cao Y, Colella NS, Liang Y, Bredas J-L, Houk KN, Briseno AL (2015). Unconventional, chemically stable, and soluble two-dimensional angular polycyclic aromatic hydrocarbons: from molecular design to device applications. Acc Chem Res.

[27] Shao X, Chunfa Xu, Long Lu, Shen Q (2015). Shelf-stable electrophilic reagents for trifluoromethylthiolation. Acc Chem Res.

[28] Prabakaran A, Vijayakumar V, Radhakrishnan N, Chidambaram R, Muthu S (2020). Experimental and quantum chemical computational analysis of novel N4, N4′-Dimethyl-[1, 1′-Biphenyl]-3, 3′, 4, 4′-Tetraamine. Polycycl Aromat Compd.

[29] Glossman-Mitnik D (2013). Computational study of the chemical reactivity properties of the Rhodamine B molecule. Proced Comput Sci.

[30] ElKhattabi S, Hachi M, Fitri A, Benjelloun AT, Benzakour M, Mcharfi M, Bouachrine M (2019). Theoretical study of the effects of modifying the structures of organic dyes based on N, N-alkylamine on their efficiencies as DSSC sensitizers. J Mol Model.

[31] Castro GT, Filippa MA, Sancho MI, Gasull EI, Almandoz MC (2020). Solvent effect on the solubility and absorption spectra of meloxicam: experimental and theoretical calculations. Phys Chem Liq.

[32] Wazzan NA, Obot IB, Kaya S (2016). Theoretical modeling and molecular level insights into the corrosion inhibition activity of 2-amino-1, 3, 4-thiadiazole and its 5-alkyl derivatives. J Mol Liq.

[33] Idumah CI, Hassan A, Ihuoma DE (2019). Recently emerging trends in polymer nanocomposites packaging materials. Polym Plast Technol Mater.

[34] Kapuria S, Bhattacharyya M, Kumar AN (2008). Bending and free vibration response of layered functionally graded beams: a theoretical model and its experimental validation. Compos Struct.

[35] Agwupuye JA, Louis H, Unimuke TO, David P, Ubana EI, Moshood YL (2021). Electronic structure investigation of the stability, reactivity, NBO analysis, thermodynamics, and the nature of the interactions in methyl-substituted imidazolium-based ionic liquids. J Mol Liq.

[36] Yu Y, Wang Y, Lin Ke, Naiyin Hu, Zhou X, Liu S (2013). Complete Raman spectral assignment of methanol in the C-H stretching region. J Phys Chem A.

[37] Louis H, Gber TE, Asogwa FC, Eno EA, Unimuke TO, Bassey VM, Ita BI (2022). Understanding the lithiation mechanisms of pyrenetetrone-based carbonyl compound as cathode material for lithium-ion battery: insight from first principle density functional theory. Mater Chem Phys.

[38] Campopiano A, Olori A, Cannizzaro A, Iannò Antonino, Capone PP (2015). Quantification of tremolite in friable material coming from Calabrian ophiolitic deposits by infrared spectroscopy. J Spectrosc.

[39] Serafini P, Milani A, Tommasini M, Castiglioni C, Casari CS (2020). Raman and IR spectra of graphdiyne nanoribbons. Phys Rev Mater.

[40] Egemonye TC, Louis H, Unimuke TO, Gber TE, Edet HO, Bassey VM, Adeyinka AS (2022). First principle density functional theory study on the electrochemical properties of cyclohexanone derivatives as organic carbonyl-based cathode material for lithium-ion batteries. Arab J Chem.

[41] Ayyappan S, Sundaraganesan N, Aroulmoji V, Murano E, Sebastian S (2010). Molecular structure, vibrational spectra and DFT molecular orbital calculations (TD-DFT and NMR) of the antiproliferative drug Methotrexate. Spectrochim Acta Part A Mol Biomol Spectrosc.

[42] Giovannini T, Egidi F, Cappelli C (2020). Molecular spectroscopy of aqueous solutions: a theoretical perspective. Chem Soc Rev.

[43] Agwupuye JA, Neji PA, Louis H, Odey JO, Unimuke TO, Bisiong EA, Ntui TN (2021). Investigation on electronic structure, vibrational spectra, NBO analysis, and molecular docking studies of aflatoxins and selected emerging mycotoxins against wild-type androgen receptor. Heliyon.

[44] Karabacak M, Kose E, Atac A, Sas EB, Asiri AM, Kurt M (2014). Experimental (FT-IR, FT-Raman, UV–Vis, 1H and 13C NMR) and computational (density functional theory) studies on 3-bromophenylboronic acid. J Mol Struct.

[45] Louis H, Amodu IO, Unimuke TO, Gber TE, Isang BB, Adeyinka AS (2022). Modeling of Ca12O12, Mg12O12, and Al12N12 nanostructured materials as sensors for phosgene (Cl2CO). Mater Today Commun.

[46] Louis H, Enudi OC, Odey JO, Onyebuenyi IB, Igbalagh AT, Unimuke TO, Ntui TN (2021). Synthesis, characterization, DFT, and TD-DFT studies of (E)-5-((4, 6-dichloro-1, 3, 5-triazin-2-yl) amino)-4-hydroxy-3-(phenyldiazenyl) naphthalene-2, 7-diylbis (hydrogen sulfite). SN Appl Sci.

[47] Mao JX (2014). Atomic charges in molecules: a classical concept in modern com-putational chemistry. J Postdr Res.

[48] Reed AE, Weinstock RB, Weinhold F (1985). Natural population analysis. J Chem Phys.

[49] Rigby J, Izgorodina EI (2013). Assessment of atomic partial charge schemes for polarisation and charge transfer effects in ionic liquids. Phys Chem Chem Phys.

[50] Wada O (2004). Femtosecond all-optical devices for ultrafast communication and signal processing. New J Phys.

[51] Weinhold F, Landis CR, Glendening ED (2016). What is NBO analysis and how is it useful?. Int Rev Phys Chem.

[52] Liu X, Rand BP, Forrest SR (2019). Engineering charge-transfer states for efficient, low-energy-loss organic photovoltaics. Trends Chem.

[53] Demircioğlu Z, Kaştaş ÇA, Büyükgüngör O (2015). Theoretical analysis (NBO, NPA, Mulliken population method) and molecular orbital studies (hardness, chemical potential, electrophilicity and Fukui function analysis) of (E)-2-((4-hydroxy-2-methylphenylimino) methyl)-3-methoxyphenol. J Mol Struct.

[54] Daoud A, Cheknane A, Hilal HS, Meftah A, Benghia A (2021). Simulation of electronic and optical properties of polyene-diphenylaniline-sensitizers for perovskite n-ZnTiO3 towards efficient dye sensitized solar cells. Mater Sci Semicond Process.

