# Supplementary material for: Synthesis, spectral characterization, and theoretical investigation of the photovoltaic properties of (E)-6-(4-(dimethylamino)phenyl)diazenyl)-2-octyl-benzoisoquinoline-1, 3-dione
Source: BMC Chem. 2022 Dec 3;16(1):109. doi: 10.1186/s13065-022-00896-w (PMC9719173; doi:10.1186/s13065-022-00896-w)
Supplement: Supplementary file 1 — Additional file 1: Table S1. Different ADCH, CHELPG, MPA and NPA charges calculated for the studied compound. Fig. S1. Experimental FTIR Spectrum. Fig. S2. Theoretical FTIR Spectrum. Fig S3. Experimental 1HNMR Spectrum. Fig. S4. Theoretical 1HNMR Spectrum. [file 13065_2022_896_MOESM1_ESM.docx]

**Synthesis, Spectral Characterization, and Theoretical Investigation of the Photovoltaic Properties of (*E*)-6-(4-(dimethylamino)phenyl)diazenyl)-2-octyl-benzoisoquinoline-1, 3-dione**

**Mbang I. Ofem^1, 2^, Hitler Louis^2, 3^, John A. Agwupuye^2, 3^*, Umar S. Ameuru^4*^, Joseph O. Odey^2, 3^, Gloria C. Apebende^2,3^, Terkumbur E. Gber^2, 3^, Neksumi Musa^5^, and Ayi A. Ayi^3, 6*^**

*^1^Department of Chemistry, Faculty of Physical Sciences, Cross River University of Technology, Calabar, Nigeria.*

*^2^Computational and Bio-Simulation Research Group, University of Calabar, Calabar, Nigeria.*

*^3^Department of Pure and Applied Chemistry, Faculty of Physical Sciences, University of Calabar, Calabar, Nigeria.*

*^4^Department of Polymer and Textile Engineering, Ahmadu Bello University, Zaria, Nigeria.*

*^5^Department of Environmnetal Sciences, Sharda University, India*

*^6^Inorganic Materials Research Laboratory, Department of Pure and Applied Chemistry, Faculty of Physical Sciences, University of Calabar, Calabar, Nigeria.*

**^*^Corresponding authors:** [**agwupuyejohn@yahoo.com**](about:blank)**:** and **a.anyama@unical.edu.ng**

**Table S1. Different ADCH, CHELPG, MPA and NPA charges calculated for the studied compound.**

| S / No | Atoms | ADCH | CHELPG | MPA | NPA |
| --- | --- | --- | --- | --- | --- |
| 1 | C | -0.0903 | -0.1427 | -0.1331 | -0.22895 |
| 2 | C | 0.1168 | 0.2197 | 0.2403 | 0.15288 |
| 3 | C | -0.0672 | -0.0222 | 0.0991 | -0.05508 |
| 4 | C | -0.0039 | 0.1181 | 0.0503 | -0.01168 |
| 5 | C | -0.0276 | -0.1874 | 0.0275 | -0.12990 |
| 6 | C | -0.0861 | -0.0252 | -0.2074 | -0.16026 |
| 7 | H | 0.1184 | 0.0623 | 0.1644 | 0.26020 |
| 8 | H | 0.1089 | 0.0969 | 0.1654 | 0.26396 |
| 9 | C | -0.0892 | -0.0567 | -0.1705 | -0.17761 |
| 10 | C | -0.0233 | -0.1589 | 0.0058 | -0.14029 |
| 11 | H | 0.1368 | 0.1054 | 0.1747 | 0.26882 |
| 12 | C | -0.0912 | -0.0089 | -0.1835 | -0.15697 |
| 13 | C | -0.1026 | -0.1178 | -0.1341 | -0.24050 |
| 14 | H | 0.1356 | 0.0842 | 0.1708 | 0.26627 |
| 15 | H | 0.1367 | 0.1098 | 0.1429 | 0.24765 |
| 16 | C | 0.2177 | 0.5355 | 0.5932 | 0.71306 |


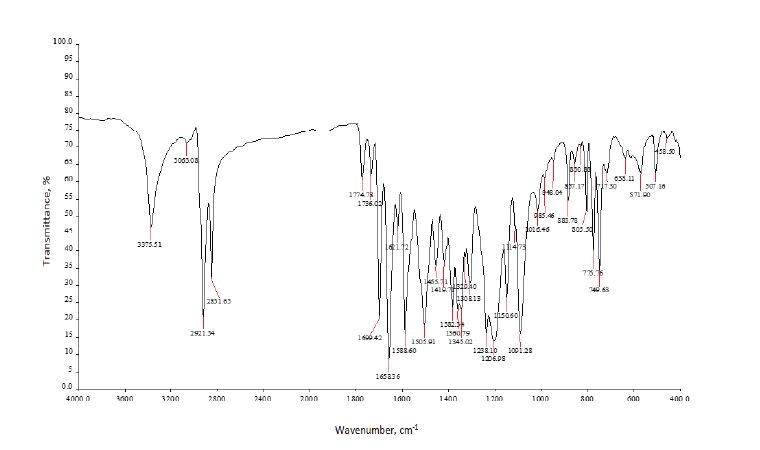


**Fig. S1. Experimental FTIR Spectrum**


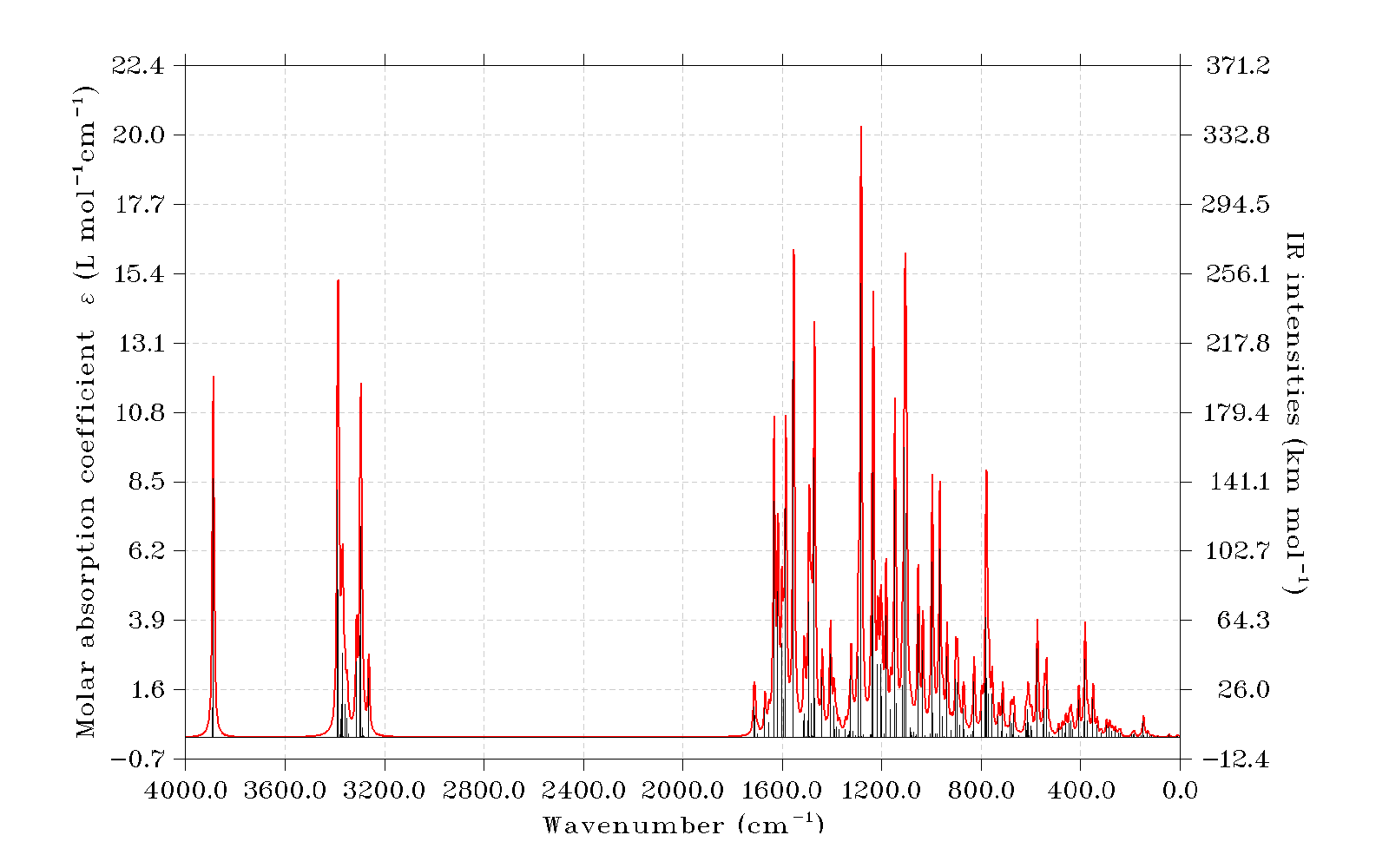


**Fig. S2. Theoretical FTIR Spectrum**


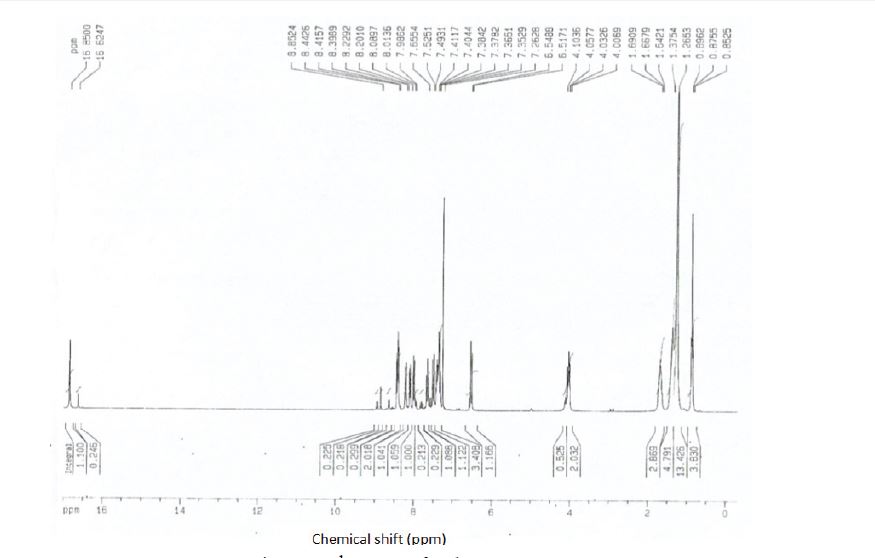


**Fig S3. Experimental 1HNMR Spectrum**


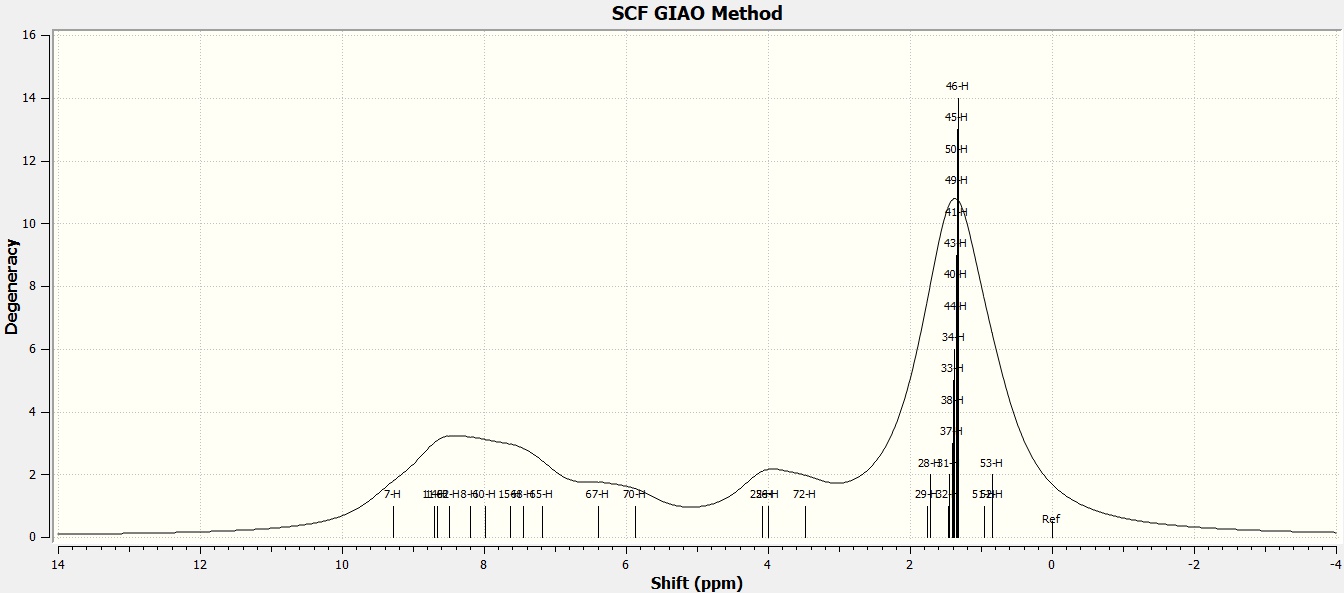


**Fig S4. Theoretical 1HNMR Spectrum**
